# Disentangling the neurobiological bases of temporal impulsivity in Huntington's disease

**DOI:** 10.1002/brb3.3335

**Published:** 2024-03-07

**Authors:** Helena Pardina‐Torner, Audrey E. De Paepe, Clara Garcia‐Gorro, Nadia Rodriguez‐Dechicha, Irene Vaquer, Matilde Calopa, Jesus Ruiz‐Idiago, Celia Mareca, Ruth de Diego‐Balaguer, Estela Camara

**Affiliations:** ^1^ Cognition and Brain Plasticity Unit Bellvitge Biomedical Research Institute (IDIBELL) Barcelona Spain; ^2^ Hestia Duran i Reynals Hospital Duran i Reynals, Hospitalet de Llobregat Barcelona Spain; ^3^ Departament de Psicologia Clínica i de la Salut Universitat Autònoma de Barcelona Barcelona Spain; ^4^ Movement Disorders Unit, Neurology Service Hospital Universitari de Bellvitge Barcelona Spain; ^5^ Department of Psychiatry and Forensic Medicine Universitat Autònoma de Barcelona Barcelona Spain; ^6^ Hospital Mare de Deu de la Mercè Barcelona Spain; ^7^ Department of Cognition, Development and Education Psychology Universitat de Barcelona Barcelona Spain; ^8^ Institute of Neurosciences Universitat de Barcelona Barcelona Spain; ^9^ ICREA (Catalan Institute for Research and Advanced Studies) Barcelona Spain

**Keywords:** delay discounting, diffusion MRI, Huntington's disease, impulsivity, white matter

## Abstract

**Background:**

Despite its impact on daily life, impulsivity in Huntington's disease (HD) is understudied as a neuropsychiatric symptom. Our aim is to characterize temporal impulsivity in HD and to disentangle the white matter correlate associated with impulsivity.

**Methods:**

Forty‐seven HD individuals and 36 healthy controls were scanned and evaluated for temporal impulsivity using a delay‐discounting (DD) task and complementary Sensitivity to Punishment and Sensitivity to Reward Questionnaire. Diffusion tensor imaging was employed to characterize the structural connectivity of three limbic tracts: the uncinate fasciculus (UF), the accumbofrontal tract (NAcc‐OFC), and the dorsolateral prefrontal cortex connectig the caudate nucleus (DLPFC‐cn). Multiple linear regression analyses were applied to analyze the relationship between impulsive behavior and white matter microstructural integrity.

**Results:**

Our results revealed altered structural connectivity in the DLPC‐cn, the NAcc‐OFC and the UF in HD individuals. At the same time, the variability in structural connectivity of these tracts was associated with the individual differences in temporal impulsivity. Specifically, increased structural connectivity in the right NAcc‐OFC and reduced connectivity in the left UF were associated with higher temporal impulsivity scores.

**Conclusions:**

The present findings highlight the importance of investigating the spectrum of temporal impulsivity in HD. As, while less prevalent than other psychiatric features, this symptom is still reported to significantly impact the quality of life of patients and caregivers. This study provides evidence that individual differences observed in temporal impulsivity may be explained by variability in limbic frontostriatal tracts, while shedding light on the role of sensitivity to reward in modulating impulsive behavior through the selection of immediate rewards.

## Graphical abstract

This study investigates individual differences in temporal impulsivity by using a delay discounting task and, its relationship with white matter connectivity. Our findings reveal significant alterations in the microstructure of key tracts of interest, including the right DLPF‐Ccn, bilateral uncinate fasciculus and the left accumbo‐frontal tract, in individuals with HD. Furthermore, we observed that variability in the structural connectivity in specific tracts is associated with individual differences in temporal impulsivity.

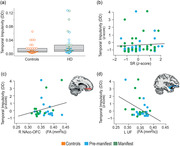

## 1 INTRODUCTION

Huntington's disease (HD) is an autosomal dominant, neurodegenerative disorder caused by an expansion of a cytosine–adenine–guanine (CAG) repeat in the *HTT* gene (MacDonald et al., 1993). Degeneration results in atrophy of the basal ganglia and cerebral cortex and disruption of the corticostriatal networks (Cepeda et al., 2007). This leads to progressive motor and cognitive deficits and behavioral abnormalities (Paoli et al., 2017). Although depressive mood, apathy, perseverative behavior, or irritability are the most extensively reported and studied as neuropsychiatric symptoms in HD, problems related to poor impulse control and the consequent impulsivity are also recognizable in HD patients (Duff et al., 2010; Johnson et al., 2017; van Duijn et al., 2013). However, studies specifically addressing impulsivity in HD are relatively scarce, with most available knowledge focusing on its relationship with other neuropsychiatric features such as mania, irritability, and aggression (Martinez‐Horta et al., 2020; Paoli et al., 2017).

Impulsivity is a complex, multifaceted construct, broadly defined as the tendency to act or react prematurely, without adequate planning or estimation of the consequences of actions, often leading to failures of self‐control, including engaging in addictive and health risk behavior patterns (Moeller et al., 2001). From the different impulsivity domains, temporal impulsivity refers to the preference for smaller, immediate rewards over larger, but delayed in time (Dalley et al., 2008). This tendency arises from the natural devaluation of delayed rewards, opting for smaller, but immediate rewards rather than potentially more valuable long‐term outcomes. However, the reward system may also favor delayed rewards if the reward is worth the effort to wait by engaging cognitive control mechanisms that facilitate the inhibition of this urge.

Individuals exhibited significant differences in their representation of how rewards are valued concerning delayed outcomes (Peters & Büchel, 2011). In particular, the delay has a more pronounced effect on subjective value in cases of impulsive behaviors and impulse control disorders (Alessi & Petry, 2003; Heerey et al., 2007; Rochat et al., 2008; Szamosi et al., 2012). That is, discount rates tend to be steeper, even when the delayed discounts have high values. In addition, other motivational biases, such as enhanced sensitivity to reward (SR) and reduced sensitivity to punishment (SP), have been identified as potential factors influencing the subjective valuation of rewards associated with impulsivity (Berlin et al., 2004; Martin & Potts, 2009).

Temporal impulsivity is typically investigated using a delay‐discounting (DD) paradigm (Madden & Bickel, 2010). This task entails assessing choices between smaller, immediate monetary rewards over larger delayed rewards to identify the DD point at which the preference for immediate rewards changes to one that is delayed. High DD rates have been reported in populations with impulse control disorders, including pathological gamblers (Alessi & Petry, 2003), as well as in patients with psychiatric conditions such as schizophrenia (Heerey et al., 2007), or neurodegenerative disorders such as Alzheimer's (Rochat et al., 2008), among others.

To our knowledge, there have been limited studies investigating temporal impulsivity within the context of HD using DD procedures. Most of these studies have primarily focused on transgenic rat models of HD (El Massioui et al., 2016; Manfré et al., 2016), revealing that transgenic HD rats presented higher levels of choice impulsivity and a lack of behavioral inhibition when compared to control rats. This results, in turn, to reduced efficiency in gambling tasks and steeper DD curves. As such, the delay degrades the value of reward more rapidly, leading to a preference for smaller, more immediate rewards. However, research involving human participants using DD task is quite scarce, and findings have been partially inconsistent. For instances, one recent study found no differences in the slope of the discounting curve between HD cases and healthy control participants (McLauchlan et al., 2022). While in contrast, another study observed a preference for immediate rewards among patients with manifested HD when compared with healthy controls, with no significant differences observed in HD individuals in the premanifest phase or the control group (El Haj et al., 2023). Overall, more studies with bigger samples are needed in order to investigate these possible effects, in HD population, in more detail.

Different neuroimaging studies have investigated the brain regions underlying DD in nonclinical populations. Aligning functional neuroimaging studies have consistently shown the involvement of the ventral striatum and the ventromedial prefrontal cortex in explaining the variability in DD (Marco‐Pallarés et al., 2010; Peters & Büchel, 2011). Specifically, activity in the ventral striatum and the ventromedial prefrontal cortex has been observed to correlate with the discounted value of future rewards in DD paradigms (Ballard & Knutson, 2009; Kable & Glimcher, 2010; Sripada et al., 2011). In the same vein, other studies have highlighted the importance of the activation of the orbitofrontal cortex (OFC) and the ventral striatum for the reward control and processing (Chumbley et al., 2014; Howard & Kahnt, 2017; Lopatina et al., 2015; McBride et al., 2006) as well as the prediction of future choices and outcomes (Leotti & Delgado, 2011; Wang et al., 2021).

The significance of these areas is largely attributed to their robust connections with other brain regions, although much remains unknown regarding their role in contributing to individual differences in temporal impulsivity. Therefore, the study of major frontostriatal white matter (WM) tracts that connect these regions, including the uncinate fasciculus tract (UF), the accumbofrontal tract (NAcc‐OFC), and the dorsolateral prefrontal cortex connecting the caudate (DLPFC‐cn), may be key. These tracts may play a crucial role in the subjective representations of the inventive value of multiple rewards and decision‐making. They have also been implicated in reward processing and impulsivity and could contribute to our understanding of the neural systems involved in DD. To the best of our knowledge, no study has explored this relationship in HD gene mutation carriers or examined the impact of the disease on ventral pathways such as the NAcc‐OFC.

In more detail, the UF is a bidirectional limbic fiber pathway that connects the anterior temporal lobe with the OFC and the amygdala. It plays a crucial role in facilitating the functional relationship between the prefrontal cortex and the mesial temporal lobe structures. The UF has been strongly related with emotional and reward processing, particularly in the context of reward‐based decisions (Camara et al., 2010; Olson et al., 2015). The NAcc‐OFC tract projects from the prefrontal cortex to the NAcc. Structural alterations in the prefrontal cortex and striatum in humans have consistently been associated with impulsive behavior (Hampton et al., 2017), as well as making risky decisions (Karlsgodt et al., 2015; Uy & Galván, 2020). In addition, a recent study (Ikuta et al., 2018) highlighted a significant positive association between impulsivity and WM integrity of the NAcc‐OFC tract. Lastly, the DLPDC‐cn tract represents a neural pathway connecting the dorsolateral prefrontal cortex and the caudate nucleus. The tract itself is associated with executive function (Crowley et al., 2021; Thompson et al., 2002), reward processing (Yuan et al., 2017), and emotional regulation (Davidson et al., 2000). Additionally, the DLPFC has also been implicated in multiple aspects of reward‐based decision‐making (i.e., reward processing), such as encoding and updating the subjective value of a reward, based on context information like reward availability and possible outcomes (Brevers et al., 2023; George & Koob, 2010, 2013; Massi et al., 2018), as well as playing a role in an individual's reward learning ability (Overman et al., 2023).

Through this framework, the aim of the present study is to investigate the behavioral and structural connectivity mechanisms underlying the individual differences in temporal impulsivity in individuals with HD, as assessed through a DD task. To this end, we explore the relationship between levels of temporal impulsivity and WM microstructure of the NAcc‐OFC, UF, and DLPFC‐cn—three major ventral frontostriatal fiber bundles associated with reward‐based decision processing.

## 2 METHODS

### 2.1 Participants

Forty‐seven HD gene expansion carriers and 36 healthy controls matched for age, sex, and years of education participated in this study. Participant demographic and clinical information are detailed in Table 1. HD participants were all confirmed gene mutation carriers with ≥36 CAG repeats. Because neural degeneration and the resulting cognitive and psychiatric symptoms are often present long before the clinical diagnosis of HD (Paoli et al., 2017), we studied the disease as a continuum across manifest and premanifest individuals, unless otherwise specified. Twenty‐five of the gene mutation carriers were manifest HD patients, defined as those with a diagnostic confidence level (DCL) ≥4 on the Unified Huntington's Disease Rating Scale (UHDRS). Eleven of the gene mutation carriers were premanifest participants with a DCL <4. One control and two HD participants did not receive a diffusion‐weighted image scan due to claustrophobia. Furthermore, WM microstructural outliers were identified and removed as *Z*‐scores greater than |2.5|. The specific *N* is detailed for each test. None of the participants reported previous history of neurological disorder other than HD. The study was approved by the ethics committee of Bellvitge Hospital in accordance with the Helsinki Declaration of 1975. All participants signed a written declaration of informed consent.

**TABLE 1:** Sociodemographic and clinical characteristics of study participants.

	Control	Manifest	Pre‐manifest
*N* ^a^	35	25	22
Sex (F/M)	18/17	14/11	18/4
Age (years)	44.00 ± 11	50.80 ± 9.39	36.64 ± 8.7
Education (years)	12.74 ± 2.7	11.24 ± 3.27	13.72 ± 2.81; *N* = 22
UHDRS‐TMS	–	21.52 ± 12.58; *N* = 25	1.52 ± 3.0; *N* = 21
CAP	–	113.02 ± 20.71	78.71 ± 14.57
DD (impulsivity)	0.013 ± 0.015; *N* = 33	0.029 ± 0.035; *N* = 17	0.021 ± 0.031; *N* = 18
SR	6.75 ± 2.93; *N* = 33	6.42 ± 3.40; *N* = 24	6.52 ± 2.84; *N* = 19
SP	9.85 ± 5.57; *N* = 34	11.83 ± 5.82; *N* = 24	10.16 ± 7.16; *N* = 19

Data presented as mean ± standard deviation.

^a^ Number of participants listed in individual cells when differing from this number for each group. Abbreviations: CAG, cytosine–adenine–guanine; CAP, standardized CAG–Age Product; DD, delay discounting, the overall *k* from Spanish version of the original Kirby Delay‐Discounting Rate Monetary Choice Questionnaire; f, females; m, males; *N*, number of participants; SP, sensitivity to punishment from the Spanish version of the Sensitivity to Punishment and Sensitivity to Reward Questionnaire; SR, sensitivity to reward from the Spanish version of the Sensitivity to Punishment and Sensitivity to Reward Questionnaire; UHDRS‐TMS, Unified Huntington's Disease Rating Scale total motor score (Huntington Study Group, 1996).

### 2.2 Clinical evaluation

The HD group underwent the clinical UHDRS evaluation, which comprises motor, cognitive, and behavioral subscales. The standardized CAG–Age Product (CAP) score was computed as CAP = 100 × age × (CAG − 35.5)/627 (Ross et al., 2014). CAP has been used to model the effects of age and CAG length on various measures of the HD state and is assumed to reflect the effects of lifelong exposure to mutant huntingtin protein. Neurologists or neuropsychologists specialized in movement disorders carried out all clinical assessments.

### 2.3 Questionnaire measures

#### 2.3.1 DD task

To assess the individual rate of discounting reward value by delay, a Spanish version of the original Kirby Delay‐Discounting Rate Monetary Choice Questionnaire was administrated on paper. For each of the 30 items, participants had to choose between two options—a smaller reward that the participant could receive sooner (immediate) or a larger amount that could be received in the future (delayed), based on a monetary choice. Additionally, three control items that represented higher amounts at present compared to smaller amounts in the future were included.

The rate of discounting, represented by the *k‐*score, is determined by the slope of a hyperbolic function through the individual subjective value of delayed rewards. This score was automatically assessed using the methodology developed by Kaplan et al. (2016). This approach was used to obtain the overall *k* measure used in this study.

The overall *k* measure is estimated based on the response pattern to all the items in the questionnaire, where each item has an associated *k* value. First, the items are sorted based on their respective associated *k* values, from small to large. For each item, participants choose either the smaller, immediate reward or the larger, delayed reward. Inferences regarding the participant's *k* value are drawn from consistent observed shifts in preference between the smaller, immediate reward and the larger, delayed reward for the different choices.

#### 2.3.2 Sensitivity to Punishment and Sensitivity to Reward Questionnaire

A Spanish version of the Sensitivity to Punishment and Sensitivity to Reward Questionnaire (SPSRQ) was completed by participants. The SPSRQ is a self‐report questionnaire with 48 Yes/No response items composed of two scales: SP and SR. Each subscale is scored on a range from 0 to 24, with higher scores indicating a higher level of SP (selective responsiveness to fear and anxiety‐evoking stimuli) or SR (selective responsiveness to stimuli with emotional well‐being, reward, and other consummatory behaviors).

#### 2.3.3 MRI data acquisition

MRI data were acquired using a 3T whole‐body MRI scanner (Siemens Magnetom Trio; Hospital Clínic), through a 32‐channel phased array head coil. Structural images comprised a conventional high‐resolution three dimensional T1‐image (magnetization‐prepared rapid‐acquisition gradient echo sequence [MPRANGE], 208 sagittal slices, repetition time [TR] = 1970 ms, echo time [TE] = 2.34 ms, inversion time [TI] = 1050 ms, flip angle = 9°, field of view [FOV] = 256 mm, 1 mm isotropic voxel).

Diffusion‐weighted MRI data were acquired using a dual spin‐echo diffusion‐tensor imaging (DTI) sequence with GRAPPA (reduction factor of 4) cardiac gating, with TE = 92 ms, 2 mm isotropic voxels, no gap, 60 axial slices, FOV = 23.6 cm. In order to obtain the diffusion tensors, diffusion was measured along 64 noncollinear directions, using a single b‐value of 1500s/mm (Cepeda et al., 2007) interleaved with 9 non‐diffusion (b = 0) images. In order to avoid chemical shift artifacts, frequency‐selective fat saturation was used to suppress fat signal.

#### 2.3.4 Diffusion‐weighted MRI tractography analysis

The gold‐standard automated probabilistic tractography approach, Tracts Constrained by UnderLying Anatomy (TRACULA) (Mazur, 1987), was employed for the dissection of the UF. Since the NAcc‐OFC and the DLPFC‐cn are not included in the TRACULA atlas, we used a deterministic dissection approach using TrackVis for these specific tracts.

#### 2.3.5 Preprocessing of DTI data

For the analysis of the UF, DTI data were automatically processed using FreeSurfer v6.0 software (http://surfer.nmr.mgh.harvard.edu/). Specifically, head motion and eddy current correction were first performed using the FMRIB's Diffusion Toolbox in FMRIB's Software Library (FSL; http://www.fmrib.ox.ac.uk/fsl/fdt) and the gradient matrix was rotated accordingly (Leemans & Jones, 2009). Boundary‐based registration method was used for the affine intrasubject alignment between the diffusion‐weighted and anatomical images, as well as to an MNI152 template (Greve & Fischl, 2009). The diffusion tensor was then reconstructed using a standard least squares tensor estimation algorithm for each voxel and then fractional anisotropy (FA), mean diffusivity (MD), and radial diffusivity (RD) maps were calculated.

Regarding NAcc‐OFC and DLPFC‐cn tracts, fiber orientation distributions were reconstructed using a spherical deconvolution approach based on the damped version of the Richardson–Lucy algorithm implemented in StarTrack software (http://www.natbrainlab.co.uk). In particular, a combination of spherical deconvolution parameters was selected to resolve crossing and avoid spurious peaks in gray matter or cerebral spinal fluid (fixed fiber response corresponding to a shape factor of *α* = 2 × 10^−3^ mm^2^/s; 200 algorithm iterations, regularization threshold *ƞ* = 0.04, and regularization geometric parameter *v* = 8) (see Dell'Acqua et al. [2010] for further details).

Whole‐brain tractography for the NAcc‐OFC and DLPFC‐cn was next performed using a b‐spline interpolation of the diffusion tensor field and Euler integration to propagate streamlines following the directions of the principal eigenvector with a step size of 0.5 mm. Tractography was terminated when FA < 0.2 or when the angle between two consecutive tractography steps was larger than 35°. Finally, the tractography data and diffusion tensor maps were exported into TrackVis (http://www.trackvis.org) for manual dissection of the tracts.

#### 2.3.6 Tractography dissections

Virtual in vivo DTI reconstruction of the UF tract was carried out bilaterally using TRACULA (Yendiki et al., 2011). TRACULA is a gold‐standard approach for probability reconstruction of major WM brain pathways and has been previously validated in HD patients. Briefly, the algorithm estimates the posterior probability distribution by combining individual participant's local diffusion orientations, extracted from the ball‐and‐stick model of diffusion (Behrens et al., 2003), with prior information from a group of training participants in which the tracts of interest were manually labeled.

Virtual in vivo DTI dissections of the NAcc‐OFC and DLPFC‐cn tracts were carried out bilaterally in native diffusion space using two regions of interest (ROIs) approach. To dissect the NAcc‐OFC tract, fibers projecting freely from the NAcc ROI were selected and restricted to terminate in the OFC, defined following established anatomical guidelines (Karlsgodt et al., 2015). To dissect the DLPFC‐cn tract, fibers projecting from the dlPFC ROI were restricted to terminate within the caudate nucleus area, as defined using the Sallet Dorsal Frontal Connectivity Based Parcellation Atlas Clusters 5, 6, and 7 (Brodmann areas 9/46 dorsal, 9/46 ventral, and 46) (Yi et al., 2016) (see Figure S1).

The ROIs were segmented using the FSL FIRST toolbox and then registered to the individual native diffusion space, a process that involved normalizing both the structural T1‐images and FA maps using the FSL FLIRT (Jenkinson & Smith, 2001) and FNIRT (Andersson et al., 2007) algorithms.

Controls and patients were randomized for the tractography that was performed by one dissector to blind participant identity. FA, MD, and RD values were extracted for subsequent analysis of WM microstructure.

### 2.4 Statistical analyses

Statistical analyses were conducted using SPSS (v.25; SPSS Inc.). To assess differences between the HD and control groups in temporal impulsivity (*k*‐score), motivational SR and SP, as well as WM disturbance within dissected tracts, independent two‐tailed *t*‐tests and Cohen's effect size calculations were applied. To further explore the differences in temporal impulsivity between the groups, both HD patients and controls were categorized into quartiles according to their overall *k*‐score. We then compared the top quartile (indicating the highest impulsivity) between the two groups in a post hoc analysis. For tract integrity, we compared differences within the HD group using both the top and lower quartiles.

To investigate the relationship between temporal impulsivity and HD progression, we employed a one‐way ANOVA with post hoc comparisons, considering control, premanifest, and manifest groups. In addition, Pearson's correlations were used to assess a possible association between temporal impulsivity scores and CAP, a proxy for disease stage.

We investigated differences in structural connectivity between HD and controls in the selected tracts by using independent two‐tailed *t*‐tests. Specifically, WM disturbances were estimated from extracted FA, MD, and RD mean values extracted from the UF, NAcc‐OFC, and DLPFC‐cn tracts bilaterally.

Afterward, we explored whether differences in WM microstructure in the main tracts of interest could explain variability in temporal impulsivity in HD participants. Multiple linear regression analyses were performed, including temporal impulsivity as the dependent variable and the predictor variables being one of the diffusion indices (FA, MD, or RD) of the three tracts of interest. Control variables included sex, age, UHDRS motor scores, and CAP. A second multiple regression analysis was conducted to explore potential motivational bias in temporal impulsivity, adding SR and SP as predictor variables. Finally, the same regression models were applied to the control group without including the HD‐specific control variables (UHDRS motor scores and CAP) to investigate whether the observed effects were specific to HD participants or explained variability in temporal impulsivity and WM microstructure in the general population.

To account for multiple comparisons, the false discovery rate (FDR) correction (*q* = .05) was used. We controlled for six comparisons (three tracts × two hemispheres) when investigating structural connectivity effects and for the two comparisons (two models) in the regression analysis. Both raw *p*‐values (*p*) and the *p*‐adjusted FDR values (*p*‐adj) are reported.

## 3 RESULTS

### 3.1 Behavioral results

#### 3.1.1 Comparison of temporal impulsivity between HD patients and controls

**FIGURE 1:**
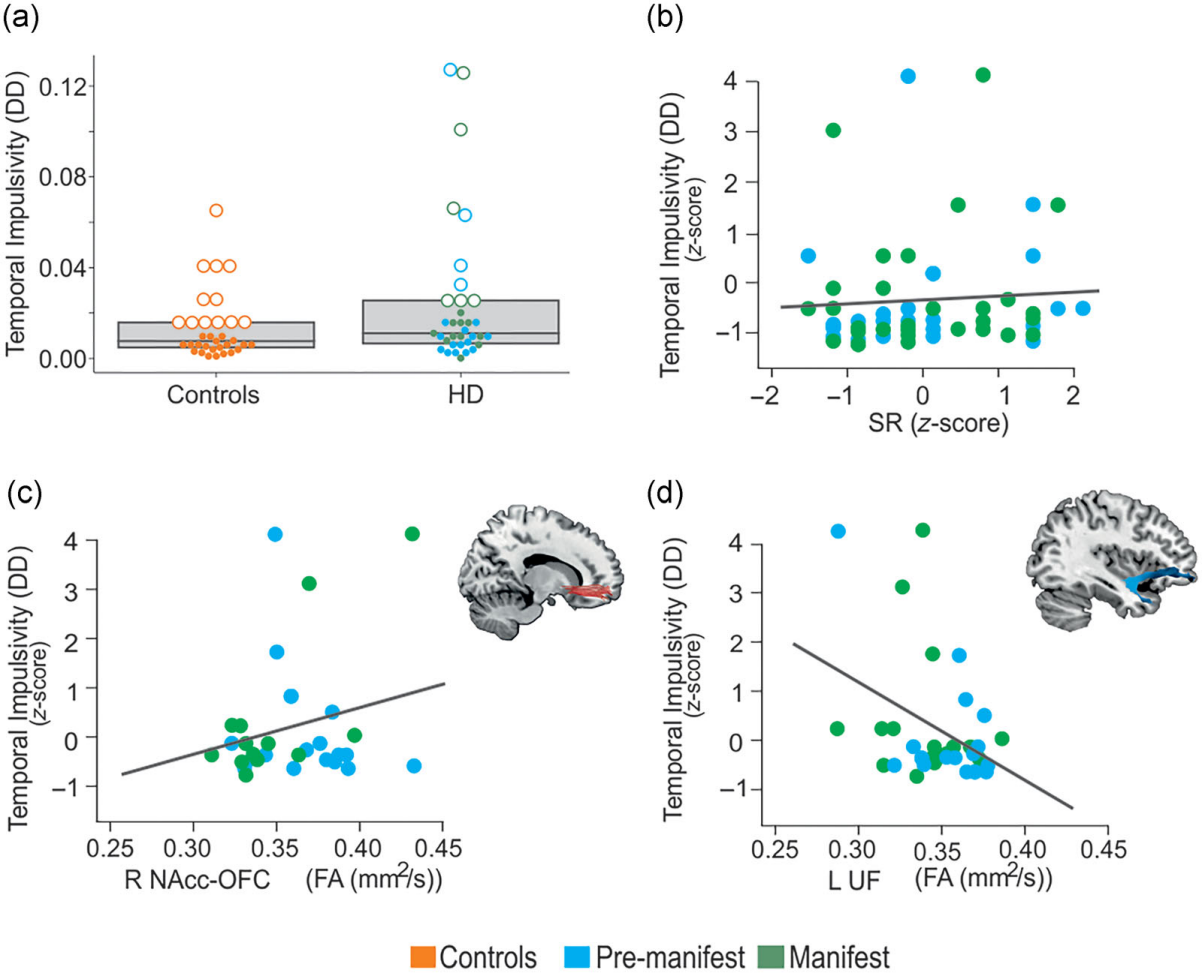
Behavioral and microstructural modulations of temporal impulsivity in Huntington's disease (HD). (A) Impulsivity scores between the controls and HD group: Scatterplot of behavioral correlates displaying the impulsivity scores between HD patients (premanifest and manifest combined) and controls. Box plots indicate the first, second (median), and third quartile limits. Individuals in the fourth quartile are marked with an empty circle, and individuals in other quartiles are marked with solid dots. (B) The relationship between impulsivity levels and SR (*β* = .007, *p* = .027). (C) The relationship between impulsivity levels and white matter disturbance in the two tracts of interest—the right NAcc‐OFC FA (*β* = .029, *p* = .002) and left UF FA (*β* = −.039, *p* = .015). The two scatterplots display the significant association obtained in the multiple regression model between impulsivity and FA in the right accumbofrontal fasciculus (NAcc‐OFC) and left uncinate fasciculus (UF). Lower FA values indicate more severe damage to white matter microstructure. Linear regression line is fit for illustration in each scatterplot. DD, delay discounting, temporal impulsivity scores; FA, fractional anisotropy; R NAcc‐OFC, right accumbofrontal tract; SR, sensitivity to reward; L UF, left uncinate fasciculus.

When comparing temporal impulsivity scores between HD patients (both premanifest and manifest, combined) (*M* = 0.025, *SD* = 0.033) and controls (*M* = 0.013, *SD* = 0.015), although we did not find statistically significant differences, there was a trend toward higher temporal impulsivity scores in HD patients (*t*(47.44) = −1.83, *p* = .074, two‐tail, Cohen's *d* = .094) (Table 1; Figure 1a). Subsequently, to study the distribution of temporal impulsivity, we performed a post hoc analysis comparing the top quartile of temporal impulsivity (indicating the highest temporal impulsivity) between the two groups. Results revealed that the top quartile of HD patients had significantly higher scores compared to the top quartile of the control group (*t*(12.11) = −2.23, *p* = .045, two‐tail, Cohen's *d* = −1.03).

#### 3.1.2 Consistency of the DD task

The DD task demonstrated excellent internal consistency among participants (Table 1). Specifically, the control group exhibited an overall task consistency of 96.84%, while the HD group showed 94.30%, indicating that participants did not engage in random responding or had unexpected response patterns.

#### 3.1.3 Group differences in temporal impulsivity and association with disease progression

We examined potential differences in temporal impulsivity scores between the three groups: premanifest (*M* = 0.021, *SD* = 0.031), manifest (*M* = 0.029, *SD* = 0.035), and control (*M* = 0.013, *SD* = 0.015), using a one‐way ANOVA with post hoc analyses. No significant between‐group differences were found (*F*(2, 65) = 2.05, *p* = .137). Likewise, no significant correlations were found when examining the association between temporal impulsivity and CAP (*r* = −.03, *p* = .87). No significant differences were found between SR (*t*(71.07) = .42, *p* = .68, *d* = .097) and SP (*t*(74.32) = −.89, *p* = .38, *d* = .78) when comparing the HD and the control group.

### 3.2 Differences in structural connectivity between HD and controls

The HD group revealed higher mean RD in both the UF and NAcc‐OFC tracts when compared with controls, demonstrating abnormal WM microstructure (Table 2). Specifically, a significant increase in RD was observed in HD individuals compared to controls in the left NAcc‐OFC (*t*(70.83) = −2.20, *p* = .031, *p*‐adj = .031, *d* = .51) and left UF (*t*(73.87) = −2.24, *p* = .028, *p*‐adj = .031, *d* = .51), as well as the right UF (*t*(73.67) = −2.24, *p* = .028, *p*‐adj = .031, *d* = −.52). Additionally, HD patients showed reduced connectivity in the right UF when compared to controls. In particular, this reduction was marked by a significant decrease in the FA values in the right UF (*t*(73.49) = 2.391, *p* = .019, *p*‐adj = .031, *d* = .55). Furthermore abnormal WM microstructure was observed in the right DLPFC‐cn in MD (*t*(71.34) = −2.712, *p* = .008, *p*‐adj = .031, *d* = −.63) when HD individuals were compared with controls (six comparisons).

**TABLE 2:** Mean FA, MD, and RD diffusion values in manifest Huntington's disease patients and controls.

			HD	*N*	Controls	*N*	*t*	*p*‐value
NAcc‐OFC	Left	FA	−0.057 ± 1.082	40	0.069 ± 0.903	33	0.539	.592
		MD	0.194 ± 1.120	40	−0.235 ± 0.786	33	−1.918	.059
		RD	0.223 ± 1.074	40	−0.270 ± 0.841	33	−2.201	.031^*^
	Right	FA	−0.039 ± 1.016	37	0.051 ± 0.995	28	0.361	.720
		MD	0.189 ± 1.045	37	−0.259 ± 0.889	27	−1.849	.069
		RD	0.192 ± 1.006	37	−0.167 ± 0.844	27	−1.548	.127
UF	Left	FA	−0.190 ± 0.904	45	0.099 ± 0.894	31	1.379	.173
		MD	0.082 ± 0.956	44	−0.224 ± 0.818	33	−1.506	.136
		RD	0.210 ± 1.042	44	−0.280 ± 0.880	33	−2.236	.028^*^
	Right	FA	−0.178 ± 0.983	44	0.322 ± 0.847	33	2.391	.019^*^
		MD	0.117 ± 0.963	44	−0.267 ± 0.805	33	−1.906	.060
		RD	0.106 ± 0.921	43	−0.325 ± 0.753	33	−2.240	.028^*^
DLPFC‐cn	Left	FA	0.028 ± 0.923	36	−0.042 ± 1.125	24	−0.254	.801
		MD	0.030 ± 0.942	35	−0.155 ± 0.960	24	−0.733	.767
		RD	−0.016 ± 0.781	36	−0.179 ± 0.821	23	−0.757	.453
	Right	FA	0.020 ± 0.999	42	−0.025 ± 1.016	33	−0.193	.847
		MD	0.213 ± 1.030	41	−0.349 ± 0.749	33	−2.712	.008^*^
		RD	0.133 ± 1.024	41	−0.244 ± 0.840	33	−1.741	.086

*Note*: MD values are given in 10^−3^ mm^2^/s and FA is given as a ratio. FA values were expected to be lower and MD values were expected to be higher in manifest Huntington's disease patients compared with controls.

Abbreviations: DLPFC‐cn, dorsolateral prefrontal cortex to caudate nucleus tract; FA, fractional anisotropy; MD, mean diffusivity; *N*, number of participants; NAcc‐OFC, accumbofrontal tract; RD, radial diffusivity; UF, uncinate fasciculus.

### 3.3 Multiple linear regression models

Multiple linear regression analyses were performed to investigate the association between temporal impulsivity and the tracts of interest in HD participants. Specifically, the more completed model taking FA values as a microstructure index, and additionally including SR and SP scores as predictors, was found to significantly explain the variance of temporal impulsivity scores (*R* ^2^ = .84, *F*(12, 9) = 4.20, *p* = .019, *p*‐adj = .038) (Figure 1B). This revealed SR (*β* = .007, *p* = .027) to be a significant contributor to the model (Figure 1C), in addition to the right NAcc‐OFC FA (*β* = .029, *p* = .002) and left UF FA (*β* = −.039, *p* = .015). However, when the SR and SP score predictors were eliminated from the model, this model was not significant (*R*2 = .60, *F*(10,13) = 1.98, *p* = .123, *p*‐adj = .123), although the specific NAcc‐OFC FA and f UF FA factors remain significant. Neither regression model showed significant results when conducted with MD or RD diffusion scores in the HD population.

Furthermore, when the multiple regression models were repeated in the control group, no regression coefficients were found to be significant in either the MD or RD models. Regarding FA, only the first model found one predictor variable to be significant, although the overall model on itself is not significant (*R* ^2^ = .68, *F*(8,7) = 1.88, *p* = .211, *p*‐adj = .320).

## 4 DISCUSSION

We aimed to investigate temporal impulsivity, as reflected by the spectrum of DD behavior, and to uncover the underlying WM differences in individuals with HD. Overall, we did not observe significant differences in temporal impulsivity (overall *k* score) between HD patients and controls. Instead, we observed a trend toward higher temporal impulsivity scores in HD patients. However, post hoc analyses showed individual differences in the distribution of temporal impulsivity among patients—particularly, in a subset of premanifest and manifest HD participants who exhibited higher temporal impulsivity scores compared to controls. This observation aligns with previous studies that have reported a lower overall prevalence of temporal impulsivity in comparison to other neuropsychiatric symptoms (Hamilton et al., 2003; Johnson et al., 2017; Paoli et al., 2017). This finding also supports a recent hypothesis suggesting that the variability in HD patient's symptomatology and pattern of neurodegeneration could be due to the existence of different profiles in HD—one focused on cognitive and motor deficits, and another on psychiatric aspects (Garcia‐Gorro et al., 2019). Consequently, it is expected that only a subset of the HD population would display higher temporal impulsivity when compared to the whole HD or a healthy population. However, it is important to note that the true prevalence of impulsivity may not be fully characterized, as this symptom is typically not evaluated as temporal impulsivity in neuropsychiatric assessments. Instead, it is often included in other neuropsychiatric categories such as disinhibition, irritability, or addictive behaviors.

The HD group exhibited disturbed microstructure in the different tracts investigated, including bilaterally the UF, the left NAcc‐OFC, and the right DLPFC‐cn, when compared to controls. This disruption was characterized by higher RD and MD values, along with reduced FA values. Notably, an increase in RD values has been associated with myelin abnormalities, while increased MD accompanied with reduced FA may represent a sign of loss of tissue architecture.

Previously, WM abnormalities in the UF have been shown in HD patients (Zhang et al., 2018). Regarding the DLPFC‐cn tract, although its diffusivity in HD individuals has not been extensively explored, a previous study found the MD of the left DLPFC‐cn tract in association with apathy. Furthermore, another study, focusing on prodromal HD population, found an increased MD (and RD) specifically in the left DLPFC‐cn (Matsui et al., 2014). However, to our knowledge, this is the first study to show altered structural connectivity in the NAcc‐OFC tract in HD. This finding is particularly interesting as the gradients of atrophy in HD start at dorsal regions of the caudate and putamen and progress to lateral regions, leaving the ventral striatum relatively unaffected until the later stages of the disease (Kassubek et al., 2004).

Neurobiologically, individual differences in temporal impulsivity have been associated with the variability in WM microstructure (Achterberg et al., 2016; Olson et al., 2009; van den Bos et al., 2014; Yu, 2012). Our findings revealed increased structural connectivity (indicated by higher FA) in the NAcc‐OFC, predicting high values of temporal impulsivity. This mirrors non‐HD studies, which also showed a significant positive relationship between WM integrity in the NAcc‐OFC tract and impulsivity (Ikuta et al., 2018). Likewise, the UF has been associated with emotion‐based evaluative processes, cognitive flexibility, and impulse control in different disorders (Olson et al., 2015; Von Der Heide et al., 2013). Our study showed a decrease in structural connectivity (indicated by lower FA) in the left UF, predicting higher temporal impulsivity scores.

This contrasts with previous delay gratification studies that associate individual variability in the UF microstructure with the ability to control the temptation of immediate rewards in favor of delayed, larger ones. For example, in healthy individuals, greater tolerance for delayed rewards, as measured by DD, was associated with increased FA in the UF in both hemispheres (Olson et al., 2009).

Regarding the DLPFC‐cn, in our results, the tract exhibited disturbed microstructure but did not show a role in explaining the individual differences of temporal impulsivity.

Previous studies have consistently shown that parallel limbic ventral corticostriatal networks may operate in a reciprocal manner (Bari & Robbins, 2013; Hampton et al., 2017). One possibility to interpret the results is that the NAcc‐OFC and UF circuits may take part in different mechanisms, with reward and cognitive control processes influencing temporal impulsivity (Bari & Robbins, 2013; Marco‐Pallarés et al., 2010; Peters & Büchel, 2011). The reward system seeks immediate rewards by estimating the incentive value, while the cognitive control system inhibits this response by predicting the consequences of the delay and/or the possible outcome (Peters & Büchel, 2011). This supports the hypothesis that these two processes operate in tandem, each represented by a parallel limbic ventral corticostriatal network (Bari & Robbins, 2013; Hampton et al., 2017; van den Bos et al., 2014).

There is limited literature on impulsivity that considers the NAcc‐OFC tract, partly due to having being only recently isolated. That being said Ikuta et al. (2018) found this tract associated with impulsive behavior and risky decision‐making (as measured using the UPPS Impulsivity Behavior Scale), reporting a significant positive correlation between its WM integrity (FA) and impulsivity in a nonclinical population. They also suggested the involvement of other corticostriatal tracts in impulsive behavior.

Regarding the DLPFC‐cn tract and its relation to temporal impulsivity, although several studies with nonclinical population highlight its role in decision‐making and link the left DLPFC activation to DD, these studies employ different temporal choice tasks (Liu et al., 2012; Weber & Huettel, 2008; Xu et al., 2009), such as the Iowa Gambling Task (He et al., 2016).

Additionally, including SR as a predictor factor revealed its mediating role in the context of temporal impulsivity. This suggests that individuals with heightened SR may be driven to elicit greater temporal impulsivity (Bari & Robbins, 2013; Moeller et al., 2001). Specifically, SR is regulated by the behavioral activation system, which in turn influences the extent to which behavior is driven by reward‐relevant stimuli. Hence, heightened SR is associated with addiction and risk‐taking behaviors, both in general and clinical populations (Kalkhoven et al., 2014).

In HD individuals, our findings also reveal diminishing valuation of reward as the delay increases. This translates to a preference for immediate monetary rewards over delayed monetary rewards, highlighting a tendency to prioritize immediate gratification over potential future outcomes. Such enhanced allure of the reward aspect even in risky or uncertain outcomes can manifest as impulsive decision‐making or a heightened susceptibility to developing gambling addiction, as has been previously suggested in HD (Kalkhoven et al., 2014; van Wouwe et al., 2016).

Another study focused on selecting advantageous decks in a gambling task proposed that the increased risky decisions in HD might results from difficulty in using feedback to learn which decks were disadvantageous, concluding that poor performance could be attributed to both impulsivity and executive dysfunction (Stout et al., 2001).

However, it is important to acknowledge certain limitations of this study. Firstly, it is important to note that the study was exploratory in nature, aiming to break a new ground in the study of temporal impulsivity in HD using the DD task, potentially modulated by the interplay between reward and cognitive control networks. A noteworthy limitation is the scarcity of literature regarding the use of a DD task in this population, with many studies conducted on animal models rather than humans, possibly misrepresenting the true expression of symptoms in human individuals. While we included control items to identify participants who may have had difficulty understanding the task, we acknowledge that these control items may not fully account for individual differences in cognitive function in our analysis. Additionally, the heterogenetic nature of HD made it challenging to account for the potential influence of medication in this study.

Furthermore, other corticostriatal frontal tracts beyond the NAcc‐OFC and UF may play a role in temporal impulsivity. Similarly, it is possible that other factors, such as alterations in time perception, mediate impulsive behavior in DD tasks (Beste et al., 2007; Cope et al., 2014; Hinton et al., 2007). Time perception has been previously observed to be affected in HD across multiple dimensions (time production, discrimination, and estimation), possibly contributing to a preference for immediate rewards (Agostino et al., 2017; Paulsen et al., 2004; Rao et al., 2014; Righi et al., 2016). Moreover, investigating the relationship between impulsivity and apathy, which has showed both positive and negative associations in other contexts (Lansdall et al., 2018), could provide clarity regarding the multidimensional nature of underlying neuropsychiatric constructs.

We highlight that the current study is focused on WM connectivity. Future investigations that integrate both WM and gray matter analyses may further provide a more comprehensive understanding of the neural correlates of impulsivity in HD. Continuing this research in larger sample size and across distinct patient populations may help to elucidate these complex questions.

Overall, these findings underscore the importance of further exploring the heterogeneous psychiatric symptomatology in HD, including less prevalent features such as temporal impulsivity, which still significantly impact the quality of life for patients and caregivers, paving the way for more personalized care approaches.

## AUTHOR CONTRIBUTIONS

Study Concept and design: Estela Camara. Analysis and interpretation of the data: Helena Pardina‐Torner, Audrey E. De Paepe and Estela Camara. Drafting of the manuscript: Helena Pardina‐Torner and Audrey E. De Paepe. Critical revision of the manuscript for important intellectual content: Helena Pardina‐Torner, Audrey E. De Paepe, Clara Garcia‐Gorro, Nadia Rodriguez‐Dechicha, Irene Vaquer, Matilde Calopa, Jesus Ruiz‐Idiago, Celia Mareca, Ruth de Diego‐Balaguer; and Estela Camara. Obtained funding: Ruth de Diego‐Balaguer; and Estela Camara.

## CONFLICT OF INTEREST STATEMENT

The authors declare no conflicts of interest.

### PEER REVIEW

The peer review history for this article is available at https://publons.com/publon/10.1002/brb3.3335.

## Supporting information

Supp Information

## Data Availability

The raw data that support the findings of this study are available from the corresponding author upon reasonable request after approval of local institutional review board.
