# Supplementary material for: Disentangling the neurobiological bases of temporal impulsivity in Huntington's disease
Source: Brain Behav. 2024 Mar 7;14(3):e3335. doi: 10.1002/brb3.3335 (PMC10918610; doi:10.1002/brb3.3335)
Supplement: Supplementary file 1 — Supp Information [file BRB3-14-e3335-s001.docx]

**Supplementary material:**

**Supplementary Figure 1**

**
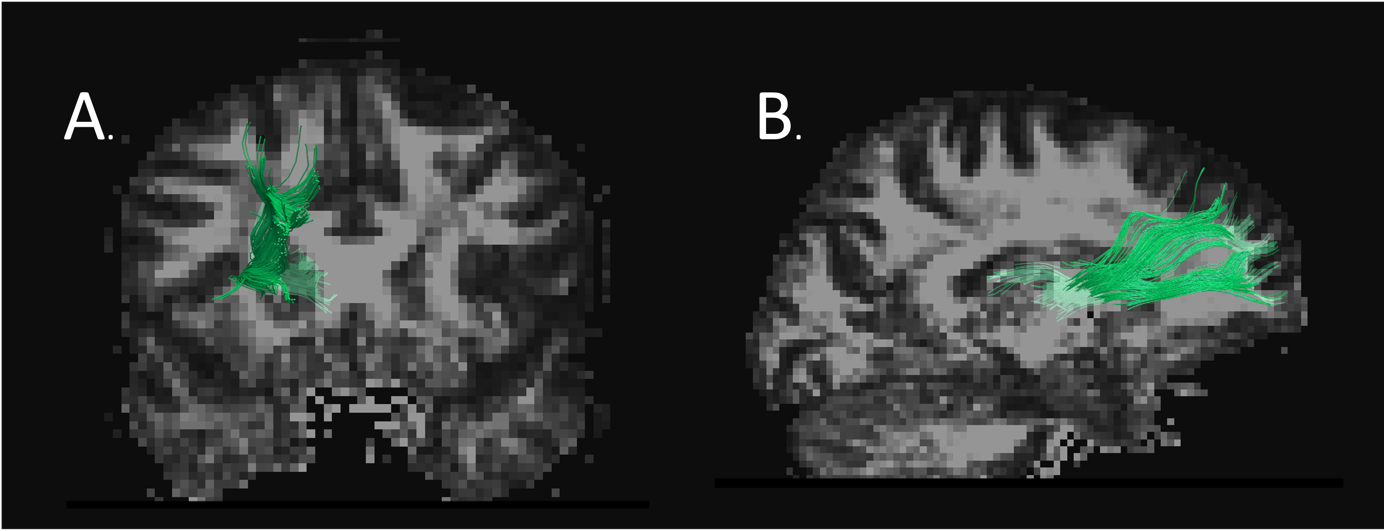
**

**Supplementary Figure 1.** In vivo coronal (A) and sagittal (B) plane dissection of the R DLPFC-cn tract (right dorsolateral prefrontal cortex to caudate nucleus) for one participant. Dissection using a deterministic approach through TrackVis.
